# P-406. Implementation of an Initial Specimen Blood Culture Diversion Device to Reduce Blood Culture Contamination: Lessons Learned

**DOI:** 10.1093/ofid/ofae631.607

**Published:** 2025-01-29

**Authors:** Francine Touzard-Romo, Diane Auld, Alison Deabreu, Kimberly Roberts, Gail Jackson, Whitehead Valerie, Emerald O’Rourke, Phinnara Has, Leonard Mermel

**Affiliations:** Newport Hospital, Alpert Medical School of Brown University, East Greenwich, Rhode Island; Lifespan Health System, Providence, Rhode Island; Lifespan Health System, Providence, Rhode Island; Lifespan Health System, Providence, Rhode Island; Newport Hospital, Newport, Rhode Island; Lifespan, Providence, Rhode Island; Lifespan, Providence, Rhode Island; Lifespan Health System, Providence, Rhode Island; Warren Alpert Medical School of Brown University, Providence, RI

## Abstract

**Background:**

Blood culture (BCx) contamination (BCxC) leads to unnecessary antibiotic exposure, increased hospital stay, and cost. Despite best practices, our health system’s BCxC rates remain above the 1% benchmark. Implementation of an initial specimen diversion device (ISDD) have demonstrated reduced BCxC rates. ^3,4^ We assessed the impact of the Kurin Lock® ISDD on BCxC and vancomycin utilization in adult emergency departments (EDs), intensive care and step-down units (ICU/SD).

**Figure:**
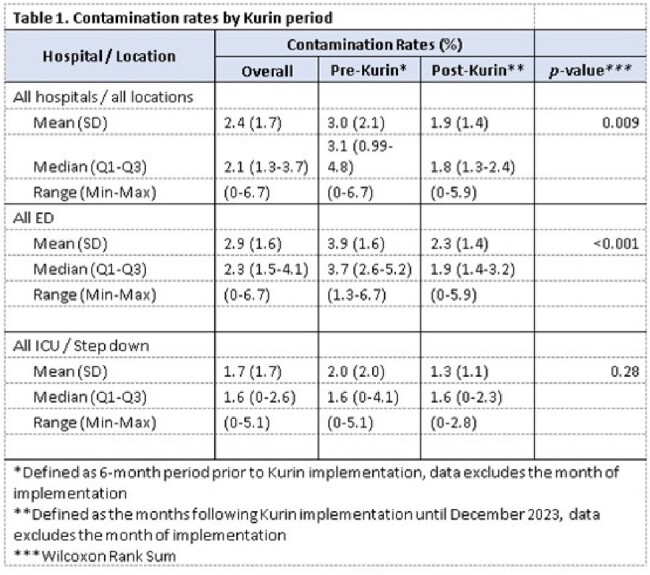

**Methods:**

The Kurin Lock® was implemented in 3 Lifespan health system hospitals (The Miriam Hospital [TMH] ED October2022; Rhode Island Hospital [RIH] and Newport Hospital [NH] EDs January 2023, and in all 3 hospital’s ICU/SD units June 2023). We included adult BCx obtained by nurses (all hospitals) or phlebotomists (NH only). BCx were drawn per protocol; growth was monitored with the BioMérieux VIRTUO System. BCxC rates were calculated dividing the number of contaminated cultures (per CDC NHSN commensal list) by the total number of BCxs/month. Mean BCxC rates prior to Kurin Lock® implementation (6 months) and after implementation (subsequent months through December 2023), excluding the month of implementation, were compared using the Wilcoxon rank-sum test. An interrupted time-series analysis was performed using binomial regression models; vancomycin days of therapy (DOT) for ’bacteremia’ was analyzed using generalized linear models.

**Figure:**
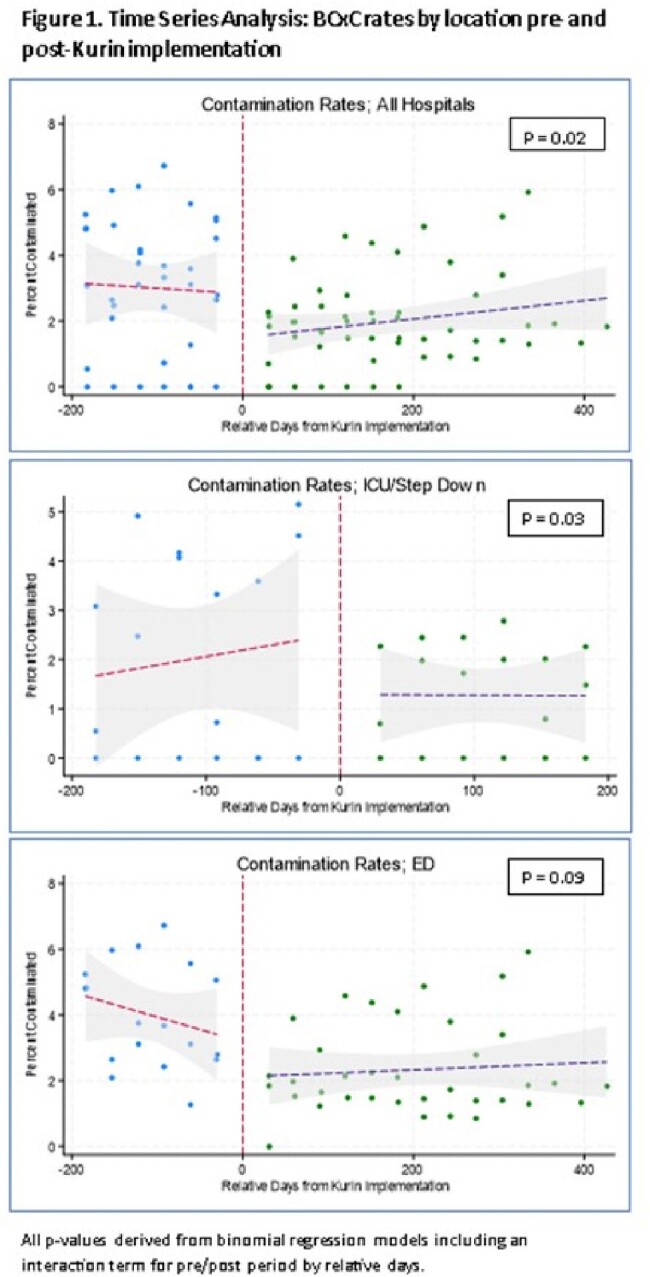

**Results:**

Overall mean BCxC rates for all hospitals and locations decreased from 3% to 1.9% after ISDD implementation (P=0.009). This decline was observed in all EDs but only statistically significant in RIH ICUs/SDs. In the time-series analysis, an abrupt 65% decline in BCxC was observed immediately after implementation in all hospitals and locations (P = 0.04). Lower BCxC rates were sustained after 200 days in the ICUs/SDs and 400 days in the EDs; however, an upward trend was observed with time. Vancomycin DOT was not significantly different pre- vs. post-Kurin Lock® implementation (41/1000 patient days vs. 37/1000 patient days, P=0.9).

**Figure:**
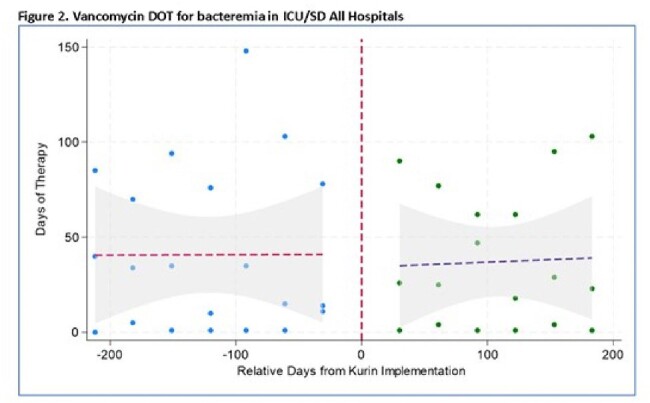

**Conclusion:**

A sustained decline in BCxC is achievable with the Kurin Lock® ISDD in academic and community hospital settings. But consistent education on best practices is key to guarantee the efficacy and cost-effectiveness of this intervention.

**Disclosures:**

**Leonard Mermel, DO, ScM**, Citius Pharmaceuticals: Advisor/Consultant|CorMedix Pharma: Advisor/Consultant|Destiny Pharma: Advisor/Consultant|Lightline Medical: Advisor/Consultant|Lightline Medical: Stocks/Bonds (Private Company)|Pristine Access Technologies: Advisor/Consultant|Pristine Access Technologies: Stocks/Bonds (Private Company)

